# ERRATUM: Nursing care in the anesthetic procedure: an integrative review

**DOI:** 10.1590/1980-220X-REEUSP-2024-ER21en

**Published:** 2024-10-07

In the article “Nursing care in the anesthetic procedure: an integrative review”, with the DOI: https://doi.org/10.1590/S0080-623420160000100020, published by the journal: “Revista da Escola de Enfermagem da USP, volume 50 (1) of 2016, p. 154–162, on page 162:

**Now read:**

## Financial support

This study was financed in part by the Coordenação de Aperfeiçoamento de Pessoa de Nível Superior – Brasil (CAPES) – Finance Code 001.

