# Supplementary material for: ERRATUM: Nursing care in the anesthetic procedure: an integrative review
Source: Rev Esc Enferm USP. 2024 Oct 7;58:e2024ER21. doi: 10.1590/1980-220X-REEUSP-2024-ER21en (PMC11524412; doi:10.1590/1980-220X-REEUSP-2024-ER21en)
Supplement: Translation

# Translation: ERRATA: Assistência de enfermagem no procedimento anestésico: revisão integrativa

No artigo “Assistência de enfermagem no procedimento anestésico: revisão integrativa”, com número de DOI: https://doi.org/10.1590/S0080-623420160000100020, publicado na Revista da Escola de Enfermagem da USP, volume 50(1) de 2016, p. 158–166, na página 166:

**Leia-se:**

## Apoio financeiro

O presente trabalho foi realizado com apoio da Coordenação e Aperfeiçoamento de Pessoal de Nível Superior – Brasil (CAPES) – Código de Financiamento 001.
